# Mandatory infectious diseases consultation and consensus approach effective at lowering antibiotic duration in uncomplicated gram-negative bacteremia

**DOI:** 10.1017/ash.2026.10892

**Published:** 2026-10-05

**Authors:** Sandra Naegele, Bianca Baik, Urmila Ravichandran, Nirav Shah, Mary Ellen Acree

**Affiliations:** 1 Department of Pharmacy, Endeavor Health, Evanston, IL, USA; 2 Department of Medicine, Endeavor Health, Evanston, IL, USA

## Abstract

This article examines the impact of Infectious Diseases (ID) practitioner consensus on gram-negative bacteremia (GNB) treatment duration. This study included 1024 adults with GNB. During the study, ID consultation for GNB was made mandatory followed by development of a GNB guideline. Antibiotic therapy was shorter after guideline development.

## Background

Gram-negative bacteremia (GNB) is a frequent cause of sepsis and is associated with a high mortality rate. In multiple studies, Infectious Diseases (ID) consultation has been shown to reduce mortality in patients with GNB.^1–3^ In recent years, several publications have demonstrated safety and efficacy of shorter courses of antimicrobial therapy for GNB than historically used.^4–7^ Ong et al found that, in addition to improving mortality, ID consultation also resulted in shorter antibiotic durations for patients with GNB, suggesting that ID practitioners are more likely to adhere to current literature on appropriate duration of therapy. This article examines the impact of gaining ID practitioner consensus on GNB management in terms of duration of antimicrobial therapy.

## Methods

This retrospective study was conducted at a 4 hospital, 828 bed health system in the northern suburbs of Chicago. Data were collected through an electronic data warehouse and via chart review from patients age 18 and older who were hospitalized with an uncomplicated bloodstream infection from any source due to *Escherichia coli*, *Klebsiella* species, or *Proteus* species between 1/1/2018–12/31/2021. Uncomplicated GNB was defined as transient, monomicrobial and lacking an abscess or other undrained focus of infection.^4,8^ Patients with planned durations of therapy exceeding 21 days were excluded, as were patients who enrolled into hospice while on antibiotics for GNB. During the study period, there were two important changes related to patients with GNB. First, beginning 9/1/2019, a policy was enacted to have a mandatory ID consultation for any patient with GNB. Second, as of 6/1/2020, a consensus guideline for GNB management was developed collaboratively among the ID physicians. The consensus guideline included recommendations on antibiotic selection, duration, and transition to oral therapy. These two events separate the study into three periods, preID consultation, transitional period after mandatory ID consultation but before ID consensus, and postID consensus. The primary outcome was days of effective antibiotic therapy. Effective therapy was defined as demonstrating in vitro activity against the pathogen identified in the blood culture.^4,5,7,8^ For organisms producing extended spectrum beta-lactamase (ESBL) enzymes, piperacillin-tazobactam was not considered effective therapy. The presence of an ESBL was identified genotypically through molecular based detection of a CTX-M gene (cobas® eplex blood culture identification panels, Roche Diagnostics, Indianapolis, Indiana) and/or phenotypically using the Kirby-Bauer disk diffusion method recommended by the Clinical and Laboratory Standards Institute.^9^

Antibiotic days of therapy were extracted manually by two pharmacists and an ID physician. Secondary outcomes included all-cause hospital mortality, length of hospital stay and 30-day readmission.

Continuous variables were described using median and interquartile range, and categorical data using frequency and prevalence. The Kruskal–Wallis rank sum test was used for continuous variables, and the χ2 test for categorical variables, to compare data between the preID consultation, transition, and postID consensus periods. To assess the impact of the ID consultation and consensus intervention on antibiotic duration, an interrupted time series analysis was conducted. The outcome was aggregated as the monthly median number of antibiotic days, selected to reduce the influence of right-skewness and extreme values. An ordinary least squares regression model was specified with terms for time (T), a postintervention indicator (D), and time since intervention (P). Time (T) was defined as a continuous monthly index across the study period. The postintervention indicator (D) was coded as 0 for all months before implementation of the intervention and 1 for all months thereafter. Time since intervention (P) was defined as the number of months elapsed after the intervention and coded as 0 for all preintervention months. This specification allowed estimation of both an immediate level change and a change in trend following the intervention. Model fit was assessed using the coefficient of determination (*R* ^2^) and the overall F-statistic, with statistical significance evaluated at a two-sided α level of 0.05. The analysis was performed using Python.

## Results

1,024 patients were included in the study. Baseline demographics were not statistically significantly different in preID consultation and postID consensus periods, including age (*P* = .518), gender (*P* = .652) and race (*P* = .480) (Table 1). The median patient age was 77.9 years and 55.1% were female. 75.3% of infections were caused by *E. coli*, followed by *Klebsiella* spp. (18.8%) and *Proteus* spp. (5.9%). Median days of effective antibiotic therapy was statistically significantly shorter in the postID consensus group (11 d) compared to the preID consultation groups (14 d) (*P* < .001) (Table 1). Patients during the transition period, during which ID was mandatorily consulted but had not yet achieved consensus, received on average 13 days of antibiotic therapy. Length of hospital stay, 30-day readmission, and in-hospital mortality were not statistically significantly different between the preID consultation and postID consensus periods in the univariate analysis.

**Table 1.** Baseline characteristics and outcomes among patients with uncomplicated gram-negative bacteremia across the preID consultation, transition and postID consensus periods Table 1 long description.

	Overall (1024)	Pre ID consultation (398)	Transition (236)	Post ID consensus (390)	*P*-value
Age in years, median [Q1,Q3]	77.9 [66.8,85.9]	76.5 [65.7,85.7]	78.3 [66.1,84.9]	78.5 [67.4,86.4]	.518
Sex at birth, n (%)					.652
Female	564 (55.1)	219 (55.0)	136 (57.6)	210 (53.7)	
Male	460 (44.9)	179 (45.0)	100 (42.4)	180 (46.2)	
Race, n (%)					.480
African American	57 (5.6)	27 (6.8)	9 (3.8)	21 (5.4)	
Asian	74 (7.2)	26 (6.5)	23 (9.7)	25 (6.4)	
Caucasian	688 (67.2)	265 (66.6)	148 (62.7)	275 (70.5)	
Declined/Unknown	4 (0.4)	2 (0.5)	1 (0.4)	1 (0.3)	
Hispanic/Latino	78 (7.6)	31 (7.8)	20 (8.5)	27 (6.9)	
Other	123 (12.0)	47 (11.8)	35 (14.8)	41 (10.5)	
Organism name, n (%)					.583
* Escherichia coli*	679 (66.3)	280 (70.4)	147 (62.3)	252 (64.6)	
ESBL *E. coli*	92 (9.0)	30 (7.5)	25 (10.6)	37 (9.5)	
*Klebsiella aerogenes*	7 (0.7)	2 (0.5)	1 (0.4)	4 (1.0)	
*Klebsiella oxytoca*	27 (2.6)	7 (1.8)	11 (4.7)	9 (2.3)	
*Klebsiella pneumoniae*	141 (13.8)	51 (12.8)	35 (14.8)	55 (14.1)	
ESBL *K. pneumoniae*	10 (1.0)	4 (1.0)	2 (0.8)	4 (1.0)	
CRE *K. pneumoniae*	2 (0.2)		1 (0.4)	1 (0.3)	
*Klebsiella variicola*	6 (0.6)	3 (0.8)	2 (0.8)	1 (0.3)	
*Proteus hauseri*	1 (0.1)		1 (0.4)		
*Proteus mirabilis*	46 (4.5)	16 (4.0)	8 (3.4)	22 (5.6)	
*Proteus species, other*	13 (1.3)	5 (1.3)	3 (1.3)	5 (1.3)	
Outcome					
LOS, median [Q1,Q3]		4.0 [3.0,6.0]	5.0 [3.0,7.0]	5.0 [3.0,7.0]	.112
Readmission, n (%)		60 (15.1)	38 (16.1)	63 (16.1)	.902
In-Hospital mortality, n (%)		1 (0.3)	1 (0.4)	1 (0.3)	.914
Median days of antibiotic therapy		14.0 [13.0,15.0]	13.0 [10.0,14.0]	11.0 [8.0,14.0]	<.001

CRE, carbapenem-resistant Enterobacterales; ESBL, extended-spectrum beta-lactamase; ID, infectious diseases; LOS, length of stay.

**Figure 1.**
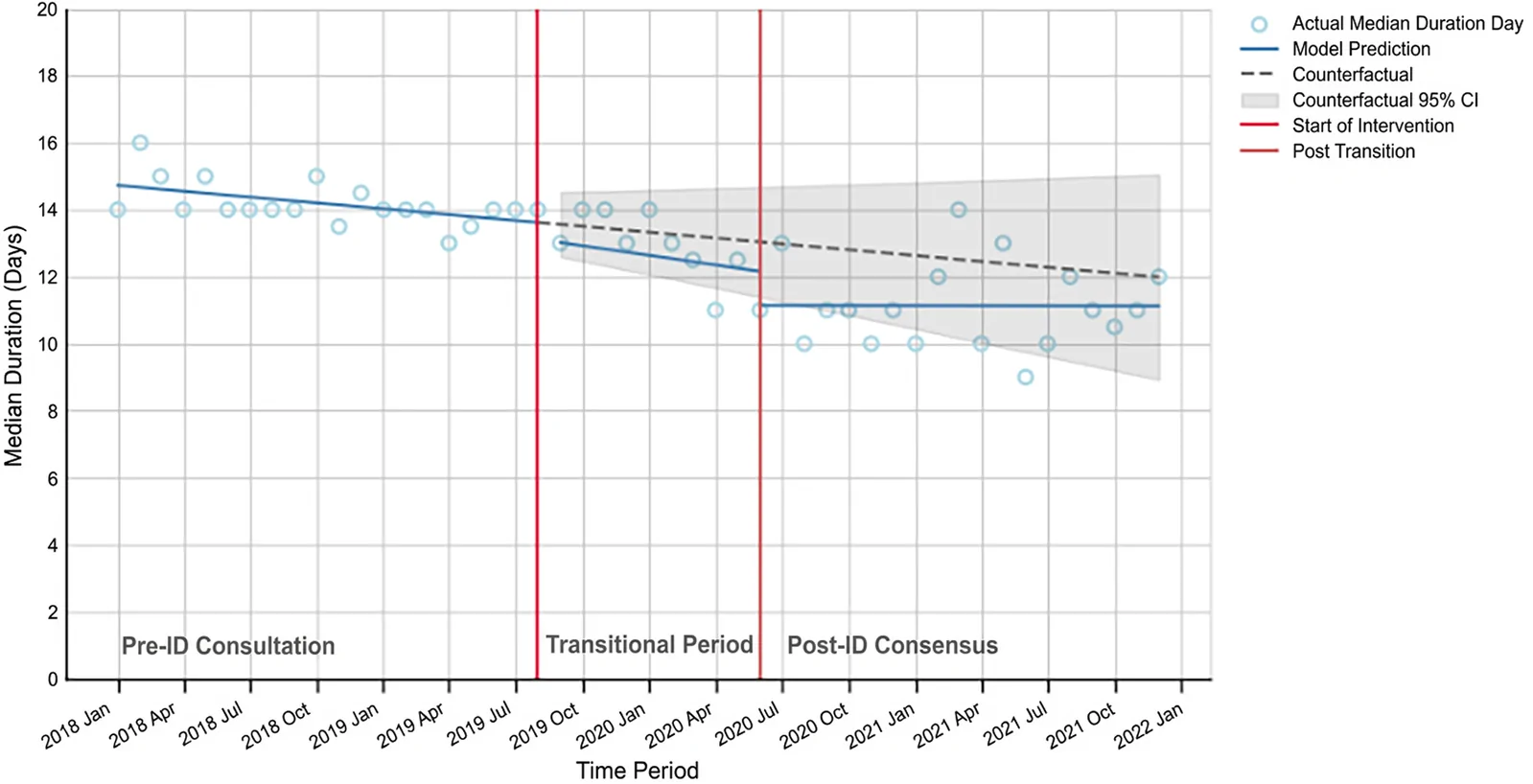
Interrupted time series of median antibiotic duration among patients with uncomplicated gram-negative bacteremia across the preID consultation, transition and postID consensus periods.

In the interrupted time series analysis, the median duration of antibiotic therapy showed a modest decreasing trend prior to the intervention (preintervention slope: β = –0.058, SE = 0.039, *P* = .141) (Figure 1); however, this change was not statistically significant. Implementation of the ID consultation and consensus guideline resulted in a statistically significant immediate reduction of 1.9 days in median antibiotic duration (β = –1.957, SE = 0.888, *P* = .034). The postintervention slope change was not statistically significant (β = 0.057, SE = 0.057, *P* = .32), indicating that the median days of therapy reached a plateau after the reduction of 1.9 days following the consensus guideline.

## Conclusions

Similar to Ong et al, this study demonstrates that mandatory ID consultation can result in shortened antibiotic duration among patients with uncomplicated GNB. However, developing consensus among ID physicians for GNB management proved more effective at shortening duration of therapy than mandatory ID consultation alone. Hospitals and health systems considering a mandatory ID consultation policy for patients with GNB should encourage their ID practitioners to develop a consensus approach to implement shorter yet effective antibiotic courses, as supported by recent literature. This study is limited by its retrospective approach and inclusion of only three gram-negative species. Further research should evaluate if ID consultation and consensus is effective at improving intravenous to oral antibiotic conversion for patients with GNB and if the impact of ID consultation and consensus is consistent among the various sources for bacteremia (genitourinary, gastrointestinal, skin/soft tissue, etc.).

## Research transparency and reproducibility

The data for this study is unavailable for review.

## Table 1. Long description

The table presents data on 1,024 patients, divided into four columns: Overall (1024), Pre ID consultation (398), Transition (236), and Post ID consensus (390). It includes rows for age, sex at birth, race, organism name, outcome, length of stay (LOS), readmission, in-hospital mortality, and median days of antibiotic therapy. Key headers include Age in years, median [Q1,Q3], Sex at birth, n (%), Race, n (%), Organism name, n (%), Outcome, LOS, median [Q1,Q3], Readmission, n (%), In-Hospital mortality, n (%), and Median days of antibiotic therapy. Notable trends include a median age of 77.9 years overall, with 55.1% of patients being female. E. coli caused 75.3% of infections. The median days of antibiotic therapy were significantly shorter in the post-ID consensus group (11 days) compared to the pre-ID consultation group (14 days).

Navigate back to Table 1..
